# Azithromycin-Induced Stevens-Johnson Syndrome in a Patient With SARS-CoV-2 and End-Stage Renal Disease

**DOI:** 10.7759/cureus.89350

**Published:** 2025-08-04

**Authors:** Jessica R Lee, Mihir K Patel

**Affiliations:** 1 Internal Medicine, University of Maryland School of Medicine, Baltimore, USA

**Keywords:** drug reaction, end-stage renal disease, macrolide, sars-cov-2, stevens-johnson syndrome

## Abstract

Stevens-Johnson syndrome (SJS) and toxic epidermal necrolysis (TEN) are a spectrum of immune-mediated mucocutaneous injuries often due to an adverse reaction to medication or infection. Numerous medications have been associated with SJS, with abacavir, allopurinol, aromatic antiepileptic drugs, minocycline, proton pump inhibitors, and sulfasalazine being the most common. Additionally, there have been several case reports of SJS associated with SARS-CoV-2 infection. The clinical presentation of SJS usually includes atypical targets or purpuric macules, along with oral mucosal involvement. Herein, we present a case of SJS with primary oral mucosal involvement in a patient with end-stage renal disease (ESRD) due to polycystic kidney disease, recent SARS-CoV-2 infection, and azithromycin exposure.

## Introduction

Stevens-Johnson syndrome (SJS) and toxic epidermal necrolysis (TEN) are the most severe of the various cutaneous adverse drug reactions. They are considered to be part of the same clinical spectrum and manifest as widespread sloughing of the skin and mucosa. The original 1993 SJS/TEN consensus definitions categorize SJS as detachment of less than 10% of the body surface area, overlap SJS/TEN as detachment between 10% and 30% of the body surface area, and TEN as detachment of over 30% of the body surface area [1]. The combined incidence of SJS, SJS/TEN overlap, and TEN is estimated to be 2-7 million cases per year [2]. Furthermore, individuals with SJS have a 10% risk of death. However, with TEN, estimated mortality climbs up to 30% or higher [3].

In 89% of cases, SJS/TEN occurs due to prescribed medications, with infections accounting for 3.7% of cases and the remaining cases being idiopathic [4]. Risk factors for developing SJS include human immunodeficiency virus (HIV), hematological malignancies, central nervous system cancers, systemic lupus erythematosus (SLE), tuberculosis, and acute and chronic kidney diseases. Certain human leukocyte antigen types are also associated with an increased risk of SJS/TEN [2,4-6]. More than 200 different medications have been linked to SJS/TEN. The most common agents include sulfonamide antibiotics (sulfamethoxazole, sulfasalazine, sulfadiazine), anticonvulsants (phenytoin, phenobarbital, carbamazepine), beta-lactam antibiotics, nevirapine, abacavir, oxicam non-steroidal anti-inflammatory medications (meloxicam, piroxicam, tenoxicam), allopurinol, lamotrigine, tetracyclines, and fluoroquinolones [5].

SJS/TEN is a clinical diagnosis characterized by widespread blister formation and flat atypical target lesions. Although clinicians frequently focus on these skin manifestations, mucous membranes are almost always involved and can cause significant discomfort. Often, there is a prodrome of fever, malaise, cough, and rhinorrhea [6]. These clinical manifestations usually present within the first eight weeks after the introduction of the offending agent [5]. Macrolide antibiotics are widely used antibiotics with a perceived low side effect profile, but they have recently been implicated as the offending medication in SJS/TEN case reports. There have been 27 reported cases of macrolide-induced SJS/TEN, with 11 cases due to azithromycin [7]. Here, we present a case of SJS in a patient with end-stage renal disease (ESRD), recent SARS-CoV-2 infection, and azithromycin exposure.

## Case presentation

A 52-year-old female with a history of chronic kidney disease stage V due to polycystic kidney disease not requiring dialysis, recent SARS-CoV-2 infection, history of immune thrombocytopenia (ITP) with a baseline platelet count of 60,000-70,000/uL, history of breast cancer treated with lumpectomy five years ago, radiation three years ago, and currently taking tamoxifen, history of iron deficiency anemia, and history of recurrent episodes of major aphthous stomatitis in adolescence treated with intravenous steroids presented with three days of painful mouth ulcers. Her medications at the time included famotidine, ferrous sulfate, lisinopril, sevelamer carbonate, tamoxifen, and omega-3 fatty acids. Twelve days prior to presentation, the patient experienced fevers, chills, sore throat, and a dry cough. At that time, she visited her primary care physician (PCP). She tested negative for SARS-CoV-2 and was prescribed azithromycin 500 mg a day for three days. Three days later, her symptoms worsened, prompting the prescription of an additional course of azithromycin 500 mg daily by her PCP, as it was felt the initial course was not long enough. During her second azithromycin course, another SARS-CoV-2 test returned with a positive result, which was treated conservatively using Tylenol 500 mg every four hours as needed for pain and fevers. Six days later, she awoke with painful oral ulcers on her tongue and the roof of her mouth. Her PCP felt this was another episode of major aphthous stomatitis and prescribed an oral methylprednisolone dose-pack. Initially, her oral ulcers were large and erythematous. However, following corticosteroid treatment, these lesions transformed to exhibit a cottage-cheese-like appearance. Despite this intervention, her symptoms did not improve after two days, and she developed pruritic rashes on her thigh, abdomen, and back, which all appeared simultaneously. At this point, her PCP advised her to seek treatment in the emergency room.

**Figure 1:**
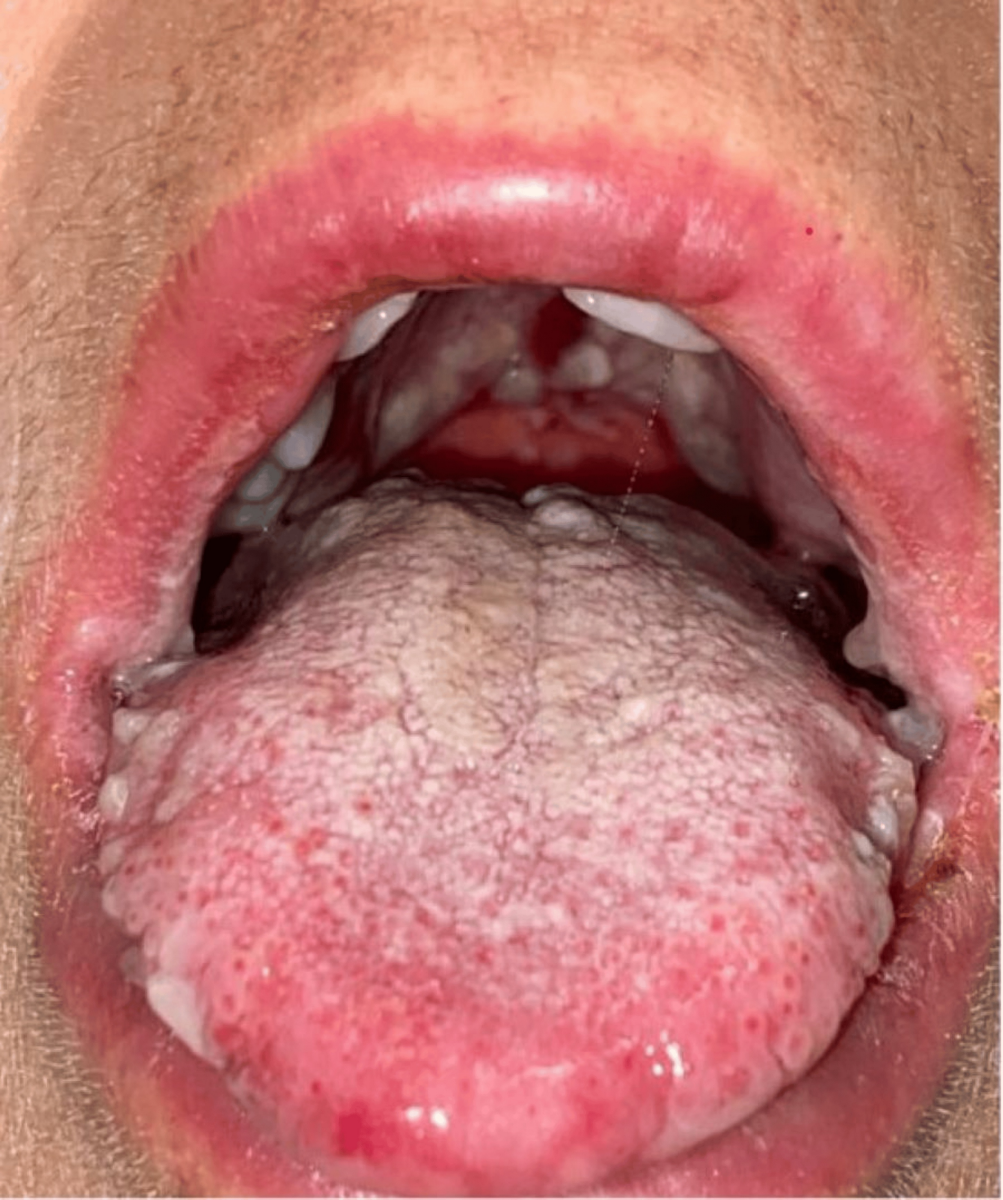
Oral ulcers on bilateral edges of the tongue.

**Figure 2:**
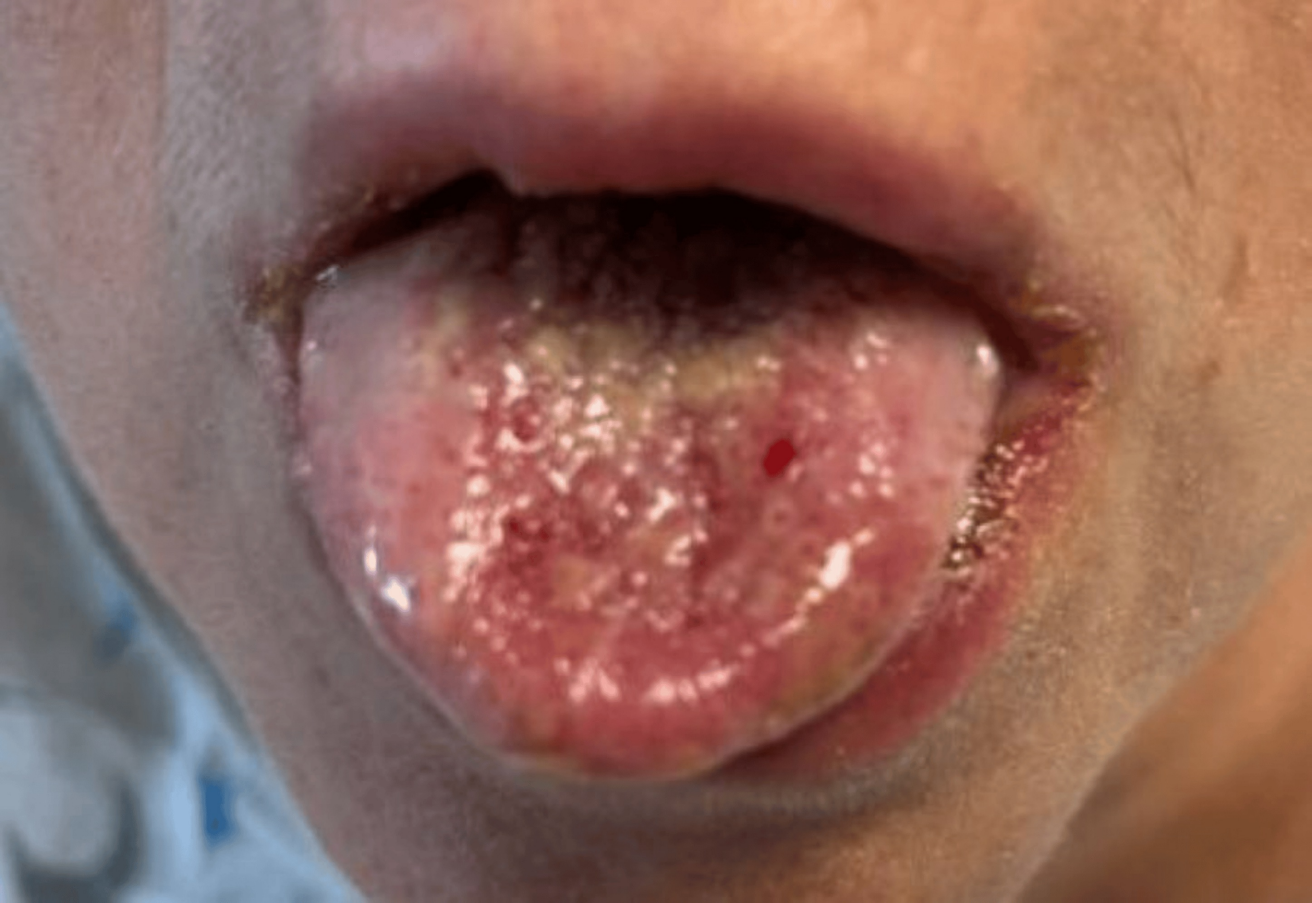
Central erosions on the tongue.

**Figure 3:**
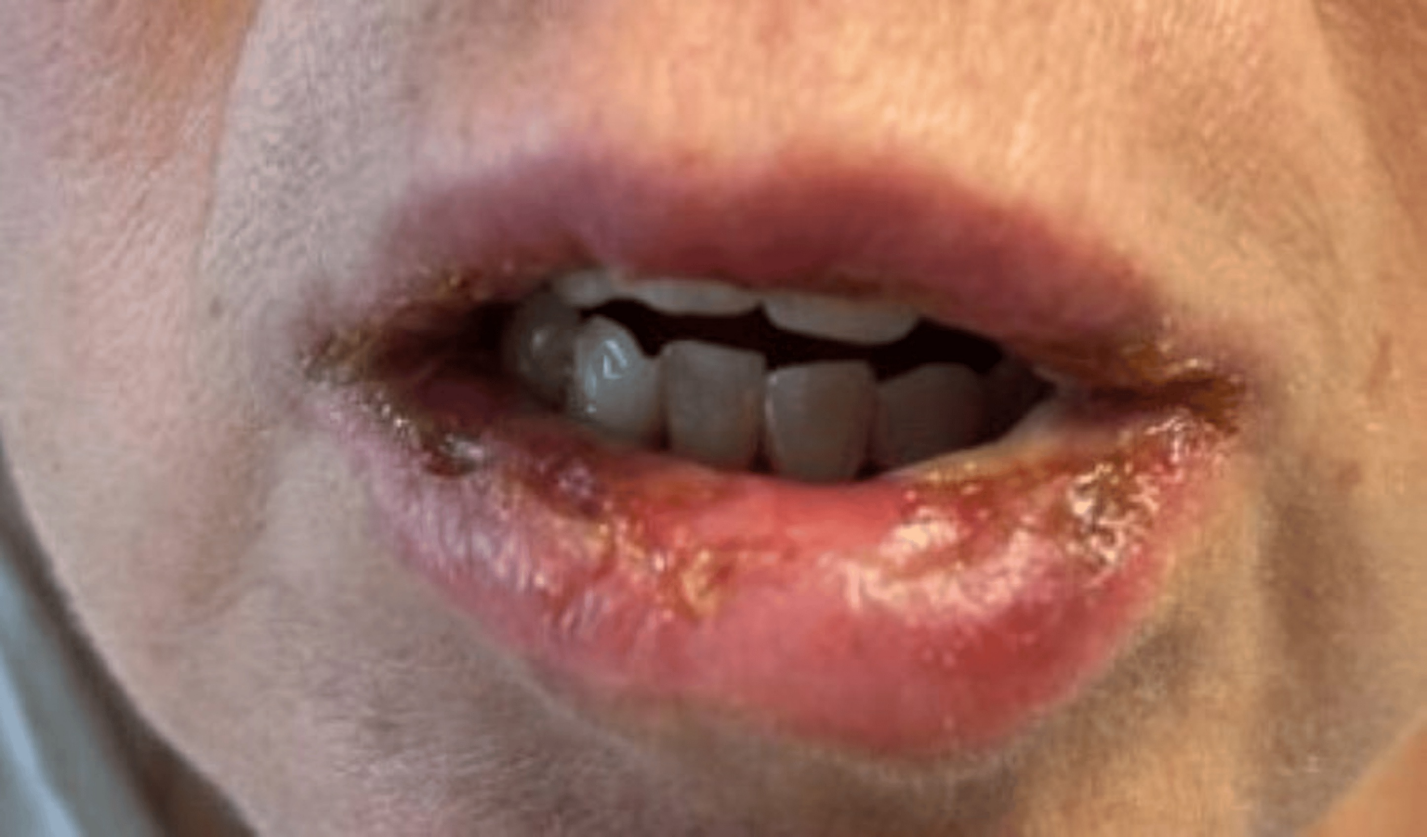
Erosions on the upper and lower vermillion lips.

**Figure 4:**
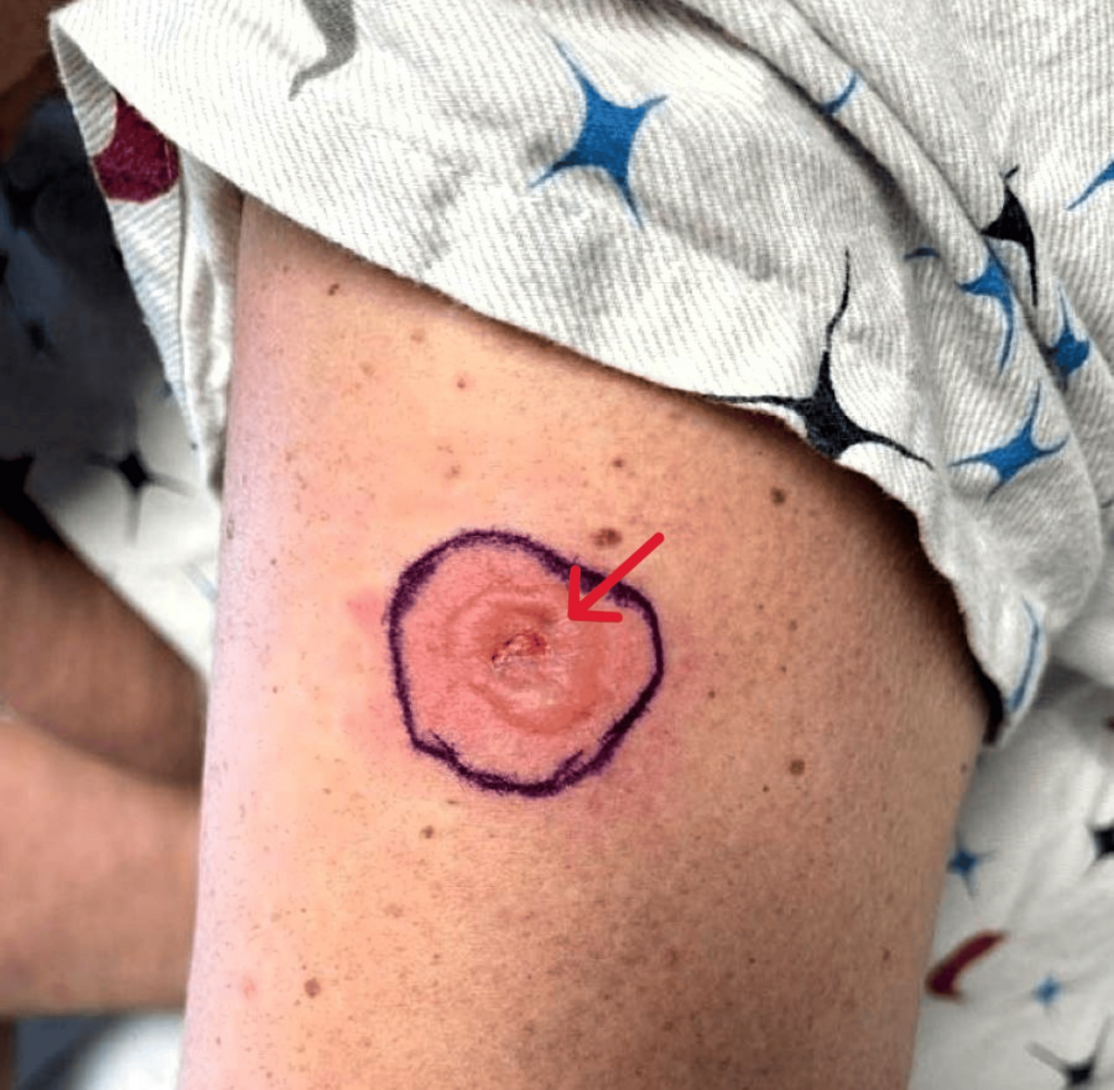
Left upper arm erythematous plaque with central bulla development.

**Figure 5:**
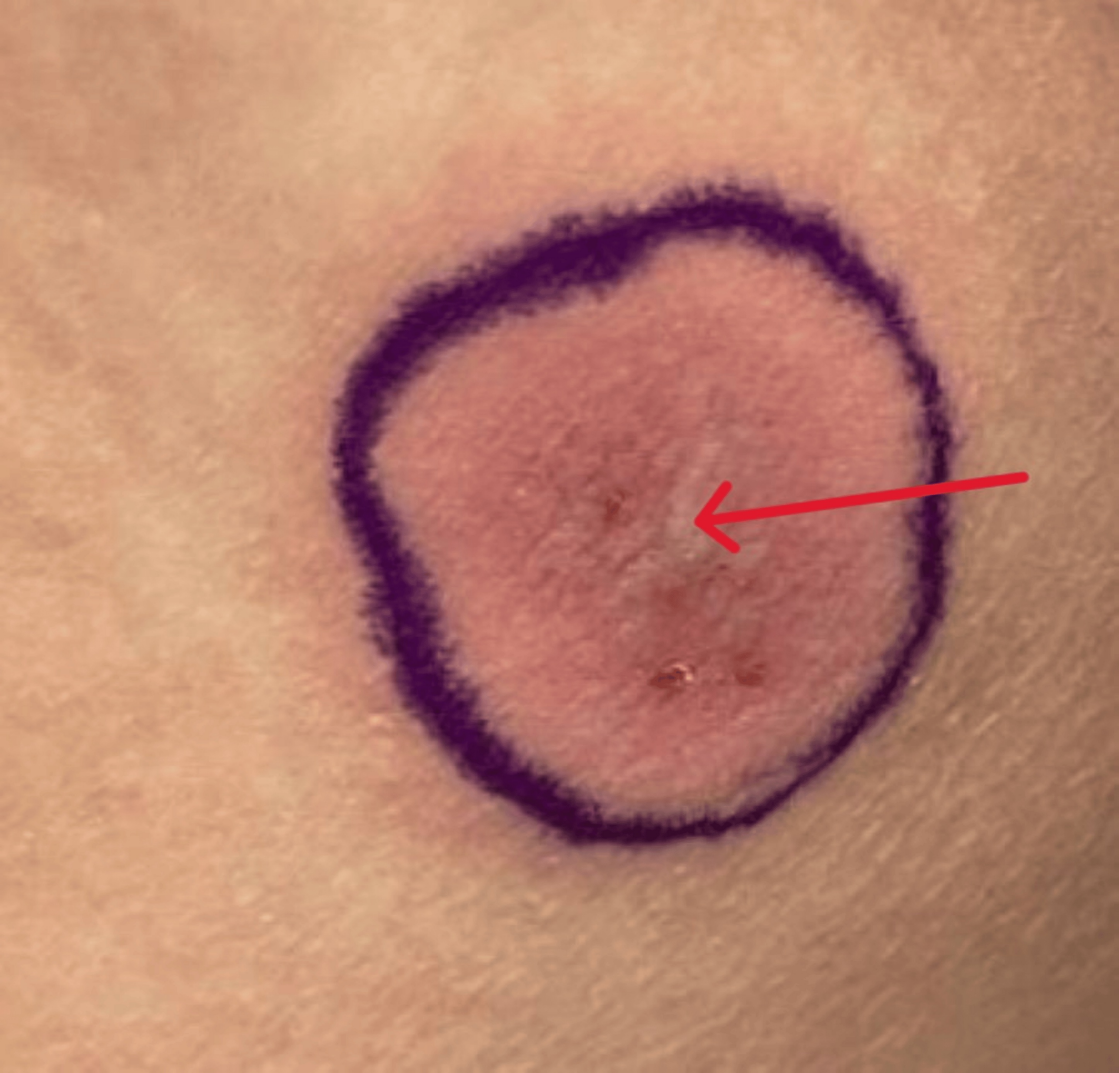
Right lower back erythematous plaque with central duskiness.

On presentation, her temperature was 97.0 F, blood pressure was 128/87 mmHg, heart rate was 94 beats/minute, and respiratory rate was 16 breaths/minute. She was noted to have oropharyngeal exudates, posterior pharyngeal erythema, bilateral conjunctival erythema with clear discharge, and an erythematous rash on the left inner thigh and left abdomen. In her oral cavity, she was found to have extensive mucosal ulceration with white exudates and sloughing (Figure 1), erosions on her tongue (Figure 2), and ulcers extending to the lips, hard palate, and buccal mucosa. She also had multiple erosions on the upper and lower vermilion lips and was unable to open her mouth widely because of the fissures at the oral commissures (Figure 3). She had an erythematous plaque on the left upper arm with central bulla development (Figure 4). She also had an erythematous plaque on the right lower back with central duskiness (Figure 5). Laboratory studies revealed a white blood cell count of 10,300/uL (reference range: 4,000-10,000/uL), hemoglobin of 14 g/dL (reference range: 12-16 g/dL), platelet count of 81,000/uL (reference range: 150,000-400,000/uL), C-reactive protein level of less than 0.5 mg/dL (reference range: <1.0 mg/dL), sedimentation rate of 10 mm/hour (reference range: 0-30 mm/hour), normal electrolytes, and normal liver enzymes. Her serum creatinine level was 5.87 mg/dL, slightly increased from her baseline of 4.30 mg/dL, but consistent with her stage V chronic kidney disease.

Our differential diagnosis included herpes simplex virus (HSV) reactivation, a SARS-CoV-2 post-infectious phenomenon, other viral exanthem, Behcet syndrome, other autoimmune mucositis, and oral candidiasis. She was initially treated with valacyclovir, which did not improve her lesions. The distribution of her lesions made a SARS-CoV-2 post-infectious phenomenon and a viral exanthem unlikely. She did not have a history of genital lesions, pathergy, or uveitis, making Behcet syndrome unlikely. Additionally, she had negative autoimmune serologies in the past, making an autoimmune etiology unlikely as well. Finally, as the lesions were more ulcerative than a plaque and did not scrape off with a tongue depressor, oral candidiasis was deemed unlikely. With the development of erythematous lesions with central bulla and duskiness, along with oral mucosal erosions, a diagnosis of SJS was made. The Alden score for azithromycin was nine, indicating that it was the causative medication [8]. The patient received high-dose intravenous corticosteroids for a duration of five days. Following this treatment, there was a slight improvement, characterized by a reduction in oral pain and an enhanced ability to tolerate oral intake. She was subsequently discharged with an 18-day prednisone taper.

Following her discharge from the hospital, the patient continued to experience complications. Her conjunctivitis took approximately one month to resolve, during which she reported a sensation akin to her eyelashes being forcibly removed. Oral and labial pain persisted, complicating her ability to consume food at home. To mitigate these issues, she avoided acidic foods, gargled with saline, and consumed nutritional shakes to optimize her dietary intake. Approximately two weeks post-discharge, her oral ulcers began to desquamate and were largely healed after an additional two weeks. The cutaneous lesions exhibited the most protracted healing process. Three months post-discharge, she retained scars on her back and arm at the sites of the initial erythematous lesions. Although these lesions have progressively healed, some scarring remains visible nine months after the initial presentation. Nonetheless, she has made a satisfactory recovery from her episode of SJS (Table 1).

**Table 1:** Clinical timeline SJS: Stevens-Johnson syndrome; TEN: Toxic epidermal necrolysis

Event	Day
Fevers, chills, sore throat, cough; azithromycin prescription	-12
Worsening symptoms; second azithromycin course	-8
Positive SARS-CoV-2 test	-6
Painful oral ulcers; conjunctivitis; methylprednisolone prescribed	-2
Development of rash on thigh, abdomen, back, arm; hospital admission	0
Valacyclovir initiated	1
SJS/TEN diagnosed - IV methylprednisolone started; valacyclovir discontinued	3
Improvement in pain and decrease in size of oral ulcers; IV methylprednisolone discontinued	8
Discharged from the hospital	9
Oral ulcers start to desquamate	23
Oral ulcers heal; conjunctivitis resolved	37
Skin lesions continue to heal - presence of scar tissue on the back and arms	90
Significant improvement - minimal scarring on the patient’s back and arms	270

## Discussion

SJS is characterized by a hyperinflammatory state, with the pathophysiology involving a dysregulated immune response. Several mechanisms contribute to the hyperinflammation seen in SJS, including immune activation, cytotoxic T-cell response, apoptosis, necrosis, and vascular endothelial activation. This immune reaction mimics a delayed type IV hypersensitivity reaction. Drugs bind to human leukocyte antigen/peptide/T-receptor complexes on keratinocytes, initiating a cascade that results in CD8+ cytotoxic T-cell and natural killer cell activation, as well as tumor necrosis factor (TNF) alpha and interferon (IFN) gamma expression. Natural killer cells release cytolytic protein/chemokine mediators (e.g., granulysin), which result in epithelial keratinocyte apoptosis. In turn, subsequent massive keratinocyte cell death causes epidermal necrolysis, leading to the hallmark blistering and erosions seen in SJS [5,7]. The immune response in SJS also affects vascular endothelial cells, leading to increased vascular permeability and tissue damage.

Azithromycin is an antibiotic used to treat conditions including pneumonia and chronic obstructive pulmonary disease (COPD) exacerbations. In the case of COPD exacerbations, azithromycin is used due to its additional anti-inflammatory properties. Azithromycin has been shown to inhibit pro-inflammatory cytokine production, inhibit neutrophil influx, and induce regular functions of macrophages [9].

A systematic review in 2021 in the International Journal of Dermatology reviewing macrolides and SJS found that 11 patients were prescribed azithromycin [7]. The time to onset of symptoms was 1-14 days, with a median of three days. Our patient’s presentation with oral lesions suggestive of SJS was six days. The acute phase of the disease lasts for approximately seven to nine days from onset before the progression halts. From then, the skin re-epithelializes over seven to 21 days. Our patient’s skin lesions were still scarring four months after azithromycin exposure. Generally, lesions re-epithelialize over two to three weeks; however, there is limited data regarding the length of time for cutaneous sequelae [10,11]. As such, we wish to use this case to highlight the importance of appropriate counseling for patients, as the resolution of SJS lesions could take more than nine months.

While azithromycin is primarily excreted by the liver, a small fraction is eliminated through the kidneys. Its pharmacokinetics may be altered in patients with ESRD due to reduced renal clearance and potential drug accumulation. The highest concentration of azithromycin in the blood increased to 61% in patients with severe renal impairment. In addition, the total drug exposure integrated over time increased to 35% in patients with severe renal impairment [12]. It is in the authors’ opinion that the patient’s ESRD and use of azithromycin led to increased accumulation of the medication in her system, which led to SJS. Moreover, chronic diseases such as diabetes, hypertension, CKD, and autoimmune conditions have been shown to increase the risk for SJS [13-15]. Furthermore, previous infection with SARS-CoV-2 has been significantly associated with an increased risk of developing autoimmune and autoinflammatory connective tissue disorders. While this case of SJS/TEN was more likely due to azithromycin exposure, recent SARS-CoV-2 infection was likely a contributing factor through cross-reactive antibodies or a release of cytokines, which further stimulated immune activation [16].

## Conclusions

This case underscores the importance of vigilance when prescribing medications to patients with ESRD, particularly those with multiple comorbidities. While azithromycin is generally considered safe, healthcare providers should be aware of the potential risks, including rare but severe adverse events like SJS. Further research is necessary to better understand the relationship between azithromycin use and immune activation in patients with CKD and to elucidate the precise mechanisms underlying the hyperinflammatory state in SJS.
